# A Game Theoretic Analysis of the Dual Function of Antibiotics

**DOI:** 10.3389/fmicb.2021.812788

**Published:** 2022-02-16

**Authors:** Ihab Hashem, Jan F.M. Van Impe

**Affiliations:** Department of Chemical Engineering, BioTeC+ & OPTEC, KU Leuven, Ghent, Belgium

**Keywords:** signaling theory, game theory, individual-based modeling, microbial communities, antibiotics

## Abstract

There are two major views toward the role of antibiotics in microbial social interactions. The classical view is that antibiotics serve as weapons, produced by a bacterial species, at a significant cost, to inhibit the growth of its competitors. This view is supported by observations that antibiotics are usually upregulated by stress responses that infer the intensity of ecological competition, such as nutrient limitation and cellular damage, which point out to a competitive role for antibiotics. The other ecological function frequently assigned to antibiotics is that they serve as signaling molecules which regulate the collective behavior of a microbial community. Here, we investigate the conditions at which a weapon can serve as a signal in the context of microbial competition. We propose that an antibiotic will serve as a signal whenever a potential alteration of the growth behavior of the signal receiver, in response to a subinhibitory concentration (SIC) of the antibiotic, reduces the competitive pressure on the signal producer. This in turn would lead to avoiding triggering the stress mechanisms of the signal producer responsible for further antibiotics production. We show using individual-based modeling that this reduction of competitive pressure on the signal producer can happen through two main classes of responses by the signal recipient: competition tolerance, where the recipient reduces its competitive impact on the signal producer by switching to a low growth rate/ high yield strategy, and niche segregation, where the recipient reduces the competitive pressure on the signal producer by reducing their niche overlap. Our hypothesis proposes that antibiotics serve as signals out of their original function as weapons in order to reduce the chances of engaging in fights that would be costly to both the antibiotic producer as well as to its competitors.

## 1. Introduction

When antibiotics were first discovered it was widely assumed that their ecological function in nature is similar to their observed effect in the laboratory settings, a “weapon” used by bacterial species to fight its competitors (Waksman and Woodruff, 1940). This was challenged by observations that the concentration of antibiotics in natural contexts, soil microbiome for example, is usually lower than their inhibiting concentrations, raising doubts about their assumed function as a weapon (Yim et al., 2006; Fajardo and Mart́ınez, 2008; Miao and Davies, 2010). Additionally, it was found that SIC of antimicrobials can induce the expression of traits that are beneficial for the recipient bacteria such as cytotoxicity, biofilm formation, and motility (Linares et al., 2006). This has led to a suggestion (Linares et al., 2006) that the assumed ecological role of antibiotics should be revised: “from weapons involved in microbial struggle for life to collective regulators of the homeostasis of microbial communities” (Linares et al., 2006). In this sense, antibiotics can be considered as an example of hormesis (Calabrese, 2004); their effect on a bacterial cell is dependent on their concentration (Linares et al., 2006). While in high concentrations antibiotics have an inhibitory effect, at lower concentrations they act as beneficial signals to the recipient bacteria (Davies et al., 2006; Yim et al., 2007). Additionally, since their concentrations in natural contexts are usually low, this endorses a higher prevalence of the later role. On the other hand, the competition sensing hypothesis (Cornforth and Foster, 2013) paints a rather different picture for the ecological function of antibiotics. The production of antibiotics is usually regulated by conditions that indicate the degree of ecological competition, such as nutrient limitation (Inaoka et al., 2003) or cellular damage (Jerman et al., 2005; Majeed et al., 2011). These regulation mechanisms strongly suggest that antibiotics are used in a competitive, not a cooperative context. Although it should be mentioned here that the usage of antibiotics in a competitive context can in some situations help in the maintenance of the stability of a consortia of different species, and thus provides community-level benefits. An interesting example of such interaction is the rock-paper-scissor dynamics where the maintenance of biological diversity is mediated by antibiotic production (Czárán et al., 2002; Baquero et al., 2019).

The competition sensing hypothesis suggests that bacteria produce antibiotics only when they infer a high degree of ecological competition, by collecting information through cues from its environment. Hence, at SICs, antibiotics serve only as a cue, through which a bacterial cell can sense ecological competition and response accordingly, whether by mounting a counter-attack or a defensive response (Bernier and Surette, 2013). Observations of these competition-oriented regulation mechanisms add again to the evidence supporting the classical view of antibiotics as weapons (Cornforth and Foster, 2015; Kallonen et al., 2017; Granato et al., 2019).

Here, we investigate the conditions necessary for a weapon to turn into a signal. The problem of weapon-signal duality is a classical problem in evolutionary game theory. The weapons of male animals can also act as signals of their fighting ability in contests over resources and access to mates. This transmission of information would allow the contestants to evaluate the gain vs. the potential cost of engaging in a fight. Hence, signaling allows both peaceful and aggressive resolution of contests. It would give rise to a peaceful resolution of uneven contests as weaker animals will run away from contests with superior ones, to the benefit of both sides of the conflict. And fighting becomes restricted to situations where the result of the fight can not be predicted in advance and the value of the resource is too high that it justifies the potential risk (Fisher, 1915; Zahavi, 1975; Hamilton and Zuk, 1982; Berglund et al., 1996). Extending these concepts to the microbial world requires its own framework, as microbes are typically thought of to have lower ability than animals to resolve their conflicts via signaling, due to their simpler regulatory networks, compared to animals' complex brains, and due to their sessile life style which limits their ability to escape a fight (Granato et al., 2019). First, we start with definitions (Maynard-Smith et al., 2003). A weapon is a substance, produced at a significant cost by a producer, to harm a competitor. On the other hand, a signal is a substance, produced at a lower cost by a sender, to communicate information for the benefit of both the sender and the receiver of the signal (Maynard-Smith et al., 2003). It is well-established that antibiotics are produced at a significant cost (Cornforth and Foster, 2013); hence antibiotics are usually only expressed at conditions where there is nutrient stress or cellular damage, in other words where there is a high level of ecological competition justifying resorting to a costly war. Now the key idea that we rely on here, is that if one is going to invest lots of resources in developing/ utilizing a highly expensive weapon, then one is also better advertising it. Signaling the capacity to do harm can lead to avoiding exercising this capacity (Berglund et al., 1996). Therefore, the following hypothesis is proposed to explain the signaling function of antibiotics: in the microbial world, an antibiotic can be used as a signal whenever an alteration in the growth behavior of the reciever reduces the competitive pressure on the producer, thus avoiding triggering the stress responses responsible for further antibiotic release. From there, three conditions can be formulated for a weapon to be used as a signal. Without loss of generality, consider a situation where there is an invader species, *I*, which has been introduced to the same niche of a resident species, *R*, potentially via migration. An antibiotic produced by *I* could act as a signal to *R* whenever the following three conditions are fulfilled:

- There is an uncertainty to whether the invader species produces the antibiotic or not.

Simply put, if the resident species has always co-evolved with the invader species in its niche, under the same mixing conditions, then there will be no point of signaling the antibiotic, as the signal will not provide new information to the resident species. There are two sources of uncertainty for a resident species in a microbial community: (i) variability in mixing conditions between the native species of a microbiome, which can bring the resident species into competition with different members of its community, and (ii) migration; new species, among them are antibiotic producing species, can migrate to the microbiome. The migrating species could or could not have the ability to inflict harm on the resident species by producing an antibiotic.

- The optimal growth strategy of the resident species would differ depending on whether the invader species is or is not a antibiotic producer.

It has been reported for example that some strains of *Escherichia coli* can respond to an initial attack by an antibiotic producer with a counterattack that targets its opponent (Basler et al., 2013). If the resident species has such strain in its neighborhood, then the antibiotic production by the resident species would not be an optimal growth strategy. On the other hand, it could otherwise be optimal to produce the antibiotic by the resident species if its competitors do not have the ability to mount such counterattack.

- The antibiotic production is costly to the producer as well.

This cost can come as (i) metabolic cost, the energy invested in antibiotic production which could have been allocated to growth, (ii) the antibiotic itself could be partly harmful to the invader species, (iii) conflict escalation with the resident species, an example is when the resident species is also capable of harming the invader species.

Hence, an antibiotic produced by *I* can be a useful signal whenever *R* can alternate it growth strategy to avoid triggering the stress responses responsible for further antibiotic production by *I*. The change in growth strategy of *R* when an invader species signaling its antibiotic production capability comes into its niche will be classified into two general categories: competition tolerance and niche segregation.

Niche segregation happens by reducing the niche overlap with the antibiotic producer. This can happen via spatial segregation, through microbial motility. When sensing the antibiotic, some bacterial species can move away from dangerous conditions (Butler et al., 2010). Another niche segregation mechanism that is highlighted here, for sessile bacterial cells, is switching to underutilized nutrients to minimize the niche overlap between competing the species (Jauri et al., 2013). While growing as a biofilm, microbial motility can be limited. Hence, the metabolic switch by the resident species could be an alternative strategy to reduce the niche overlap in the metabolic space instead of the spatial space. Alternatively, the resident species can also keep consuming the resource shared with the invader but with a change in its growth strategy that reduces the competitive stress on the invader. This class of responses will be labeled competition tolerance. Where competition tolerance refers to alternative growth strategies that can be implemented by the resident species to reduce the nutrient stress on the invader, with such strategies being characterized by a lower growth rate but better utilization of resources. Finally, it should be noted that the assumption that the invader species is a producer of an antibiotic to which the resident species is sensitive is made without loss of generality; an established population could produce antibiotic as well as a defense strategy/ signal against arriving migrant species sensitive to that antibiotic (Wiener, 1996).

## 2. Materials and Methods

### 2.1. Model

A model of a competition between two bacterial species, one that produces an antibiotic to which the other is sensitive is simulated. The model is described via the following set of equations (Bucci et al., 2011; Cornforth and Foster, 2013):


(1) $$\begin{array}{r} \frac{dI}{dt}=\left(1-fH\left(N_{th}-N\right)\right)\mu I \end{array}$$

(2) $$\begin{array}{r} \frac{dR}{dt}=\left(\mu -K_{A}A\right)R \end{array}$$

(3) $$\begin{array}{r} \frac{dA}{dt}=\alpha fH\left(N-N_{th}\right)\mu I-\beta_{A}A \end{array}$$

(4) $$\begin{array}{r} \frac{dN}{dt}=\frac{-1}{Y}\mu \left(I+R\right) \end{array}$$

(5) $$\begin{array}{r} \mu =\mu_{max}\frac{N}{N+K_{N}} \end{array}$$

With *I* (mg bacteria/l) and *R* (mg bacteria/l) as the concentrations of the invader and the resident species, respectively. *I* produces an antibiotic *A* (mg/l) by investing a fraction of its metabolic energy, *f*, in its production, and both *I* and *R* consume nutrient *N* (mg/l). The antibiotic *A* has a killing rate of *K*_*A*_ (l/mg antibiotic/h) and a decay rate that is denoted by β_*A*_ (/h). The growth dynamics are modeled after Monod dynamics with μ (/h), μ_*max*_ (/h), and *K*_*N*_ (mg/l) as the growth rate, maximum growth rate, and half saturation constant of the Monod equation, respectively. Finally, the production of the antibiotic is regulated by a stress response such that it is only initiated when the nutrient concentration is lower than a certain threshold *N*_*th*_ (mg/l), this condition is expressed by the Heaviside step function *H*(*N*_*th*_−*N*), which is equal to one only when *N* < *N*_*th*_.

### 2.2. Competition Tolerance

To model a signaling game, the invader's equation is modified such that it always produces a signal of its capability of antibiotic production by investing a small fraction of its metabolic energy, *f*_*s*_, in its production. Correspondingly, the resident species can perform a set of actions, utilizing alternative growth strategies, depending on whether a signal has been received or not. For the competition tolerance response, *R* switches from high growth rate/ low yield strategy to low growth rate/ high yield strategy based on the existence of a threat. This means that the growth dynamics of *I* and *R* will be expressed as follows:


(6) $$\begin{array}{r} \frac{dI}{dt}=\left(1-fH\left(N_{th,high}-N_{high}\right)-f_{s}\right)\mu I \end{array}$$

(7) $$\frac{dR}{dt}=((H(A-A_{s})\mu_{1}{}^{\circ}H(A_{s}-A)\mu_{2}-K_{A}A)R$$

(8) $$\begin{array}{r} \frac{dA}{dt}=\alpha fH\left(N-N_{th}\right)\mu I-\beta_{A}A \end{array}$$

(9) $$\begin{array}{r} \frac{dN}{dt}=\frac{-1}{Y}\mu I+\left(H\left(A-A_{s}\right)\frac{-1}{Y_{1}}\mu_{1}+H\left(A_{s}-A\right)\frac{-1}{Y_{2}}\mu_{2}R\right) \end{array}$$

(10) $$\begin{array}{r} \mu =\mu_{max}\frac{N}{N+K_{N}},\mu_{1}=\mu_{max,1}\frac{N}{N+K_{N}},\mu_{2}=\mu_{max,2}\frac{N}{N+K_{N}} \end{array}$$

Following Kreft (2004), we abstract a growth strategy into the combination of the maximum growth rate and growth yield, (μ_*max*_, Y), characterizing the metabolic configuration of a certain strain. For the resident species, (μ_*max*_, Y) can take values out of possible set of actions *A* = {(μ_*max*, 1_, *Y*_1_), (μ_*max*, 2_, *Y*_2_)}, with μ_*max*, 1_ and *Y*_1_ as the maximum growth rate and yield abstracting the high growth rate/ low yield strategy, μ_*max*, 2_ and *Y*_2_ on the other hand describe the low growth rate/ high yield, metabolically efficient, strategy.

To simulate a non-efficient antibiotic producer, the stoichiometric coefficient for antibiotic production by the non-efficient, cheater strain is set to α = 0.4 (mg antibiotic/mg bacteria) instead of the nominal value.

### 2.3. Niche Segregation

For the niche segregation response, the basic model is modified as follows:


(11) $$\begin{array}{r} \frac{dI}{dt}=\left(1-fH\left(N_{th,high}-N_{high}\right)-f_{s}\right)\mu_{high}I \end{array}$$

(12) $$\begin{array}{r} \frac{dR}{dt}=\left(H\left(A-A_{s}\right)\mu_{low}+H\left(A_{s}-A\right)\max\left\{\mu_{high},\mu_{low}\right\}-K_{A}A\right)R \end{array}$$

(13) $$\begin{array}{r} \frac{dA}{dt}=\alpha fH\left(N_{high}-N_{th,high}\right)\mu I-\beta_{A}A \end{array}$$

(14) $$\frac{dN_{high}}{dt}=\frac{-1}{Y}(\mu_{high}I{}^{\circ}H(A_{s}-A)H(\mu_{high}-\mu_{low}))\mu_{high}R)$$

(15) $$\frac{dN_{low}}{dt}=\frac{-1}{Y}(H(A-A_{s})\mu_{low}+$$

$$H(A_{s}-A)H(\mu_{low}-\mu_{high}))\mu_{low}R)$$

(16) $$\begin{array}{r} \mu_{high}=\mu_{max,high}\frac{N_{high}}{N_{high}+K_{N}} \end{array}$$

(17) $$\begin{array}{r} \mu_{low}=\mu_{max,low}\frac{N_{low}}{N_{low}+K_{N}} \end{array}$$

with *N*_*high*_ (mg/l) is the high value substrate, which can support a maximum growth rate of μ_*max,high*_ (1/h), while *N*_*low*_ (mg/l) is the low value substrate, with a corresponding maximum growth rate of μ_*max,low*_ (1/h). Hence, depending on the existence of a signal, the set of possible actions of *R* can be expressed as *A* = {μ_*low*_, maxμ_*high*_, μ_*low*_}. In absence of a signal, *R* consumes the nutrient which provides it with higher growth rate. If a signal of a nutrient stress regulated antibiotic producer is received, *R* switches to exclusively consume the low value nutrient, growing with a rate of μ_*low*_ (1/h).

### 2.4. Modeling Platform

All individual-based modeling simulations were carried out using MICRODIMS, an in-house IbM platform that has been developed at BioTeC+ and applied for modeling spatial microbial growth phenomena as colonies and biofilms (Verhulst et al., 2011; Tack et al., 2015, 2017). MICRODIMS is in-turn built using Repast Simphony (North et al., 2013), a multi-purpose inidvidual-based modeling toolkit, and written in Java. MICRODIMS shares the same design principles of other IbM implementations which have been applied to understand social interactions within biofilms (Picioreanu et al., 1998; Kreft et al., 2001; Xavier and Foster, 2007; Mitri et al., 2011). The main biological processes of individual cells are modeled including their growth, reproduction and death, and the behavior of the overall population emerges from their interactions. A shoving algorithm is used to prevent the overlap of the neighboring cells in the biofilm (Kreft et al., 2001). Otherwise, the cells are sessile, i.e., other types of motion (e.g., swimming, twitching, Brownian motion,...) are not included in the model. Also, the diffusion of nutrients and the antibiotic within the grid is simulated and solved using a Forward-Time-Central-Space algorithm. All the simulations were carried out for 50 times, and the mean of the results has been plotted, with a confidence interval >95% and standard deviation ≈1%. The initial nutrients concentrations were set to be 1 mg/l. All of the simulations were conducted using a 500 × 200 μ*m* grid, seeded with uniformly distributed 100 cells of each strain and carried out till the biofilm height reached 150 μ*m*. The nominal values of all the parameters are provided in Table 1.

**Table 1:** Parameter values.

**Parameter**	**Denotation**	**Value**
*f*	Fraction of energy invested in antibiotic production	0.1
*K*_*N*_	Half saturation constant	5 × 10^−4^ (mg/l)
*K*_*A*_	The antibiotic's killing rate	1.5 × 10^−4^ (l/mg antibiotic/h)
β_*A*_	The antibiotic's decay rate	10^−1^ (/h)
μ_*max*_	Maximum growth rate	1 (/h)
*Y*	Growth yield	0.7 (mg bacteria/mg nutrients)
α	The antibiotic's stoichiometric coefficient	4 (mg antibiotic/mg bacteria)
*N*_*th*_	Nutrient threshold for the antibiotic release	0.5 (mg/l)
*f*_*s*_	Fraction of energy invested in signal production	0.001
μ_*max,high*_	Maximum growth rate on the high value substrate	1 (/h)
μ_*max,low*_	Maximum growth rate on the low value substrate	0.8 (/h)
μ_*max*, 1_	Growth rate value, high growth rate strategy	1 (/h)
μ_*max*, 2_	Growth rate value, high yield strategy	0.5 (/h)
*Y*_1_	Yield value, high growth rate strategy	0.35 (mg bacteria/mg nutrients)
*Y*_2_	Yield value, low growth rate strategy	0.7 (mg bacteria/mg nutrients)

## 3. Results

### 3.1. Competition Tolerance

**Figure 1:**
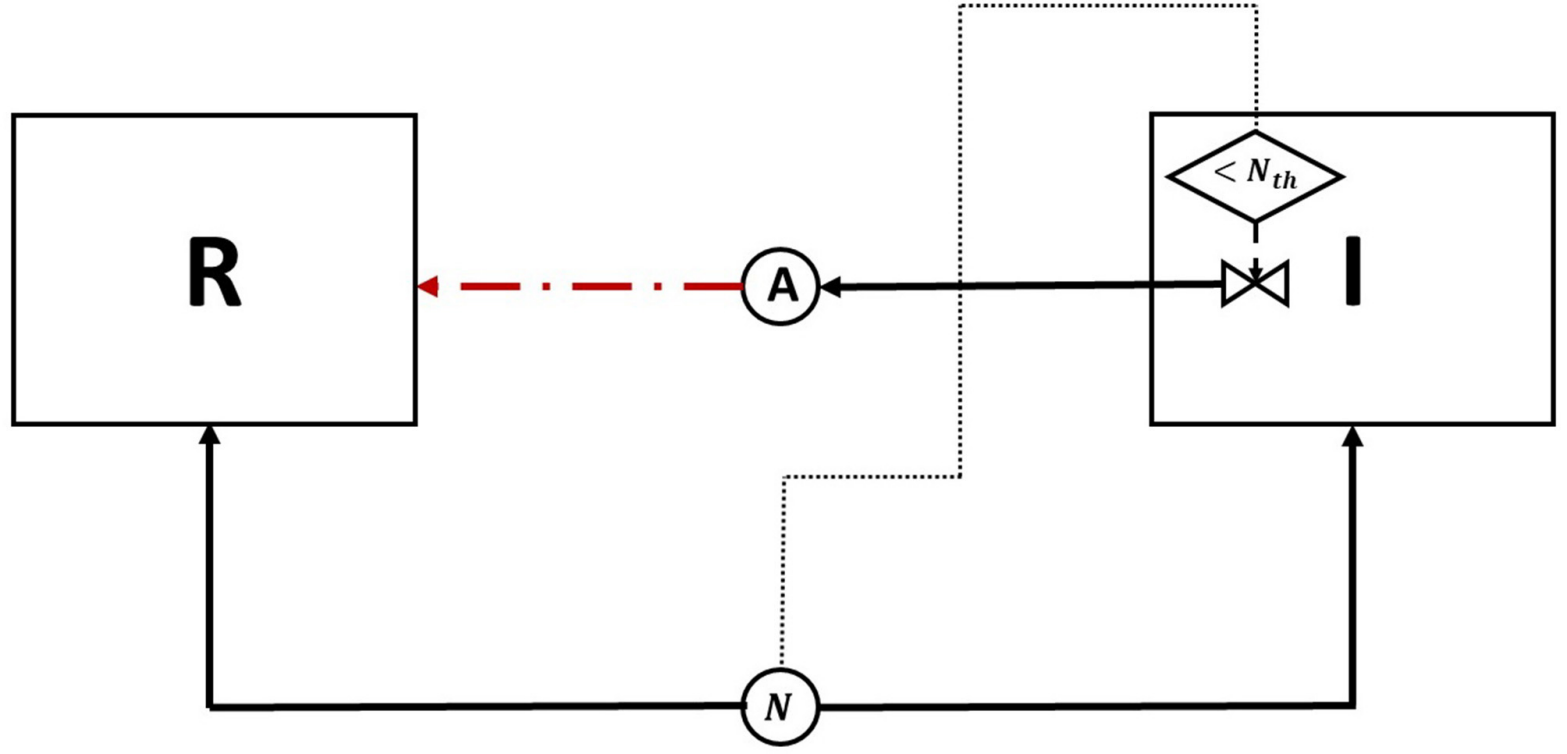
A diagram of the competition between an antibiotic producing invader vs. a resident species, competing over a single nutrient. The change in nutrient utilization strategy by the resident species in response to competition with a nutrient stress regulated invader is examined. The diagram depicts two biological species: the invader (*I*) and the resident (*R*), both enclosed in a square, and two chemical species, a nutrient (*N*) and an antibiotic (*A*), denoted by circles. The consumption of *N* by *I* and *R*, as well as the production of *A* by *I* are all denoted by solid lines. The growth inhibiting effect of *A* on *R* is denoted by a dashed line. Finally, the production of *A* by *I* is regulated by nutrient stress. This is represented by a decision flowchart where *I* senses the nutrient concentration, this action is represented by a dotted line, and it activates the production of *A* only when it falls below a threshold concentration *N*_*th*_.

**Figure 2:**
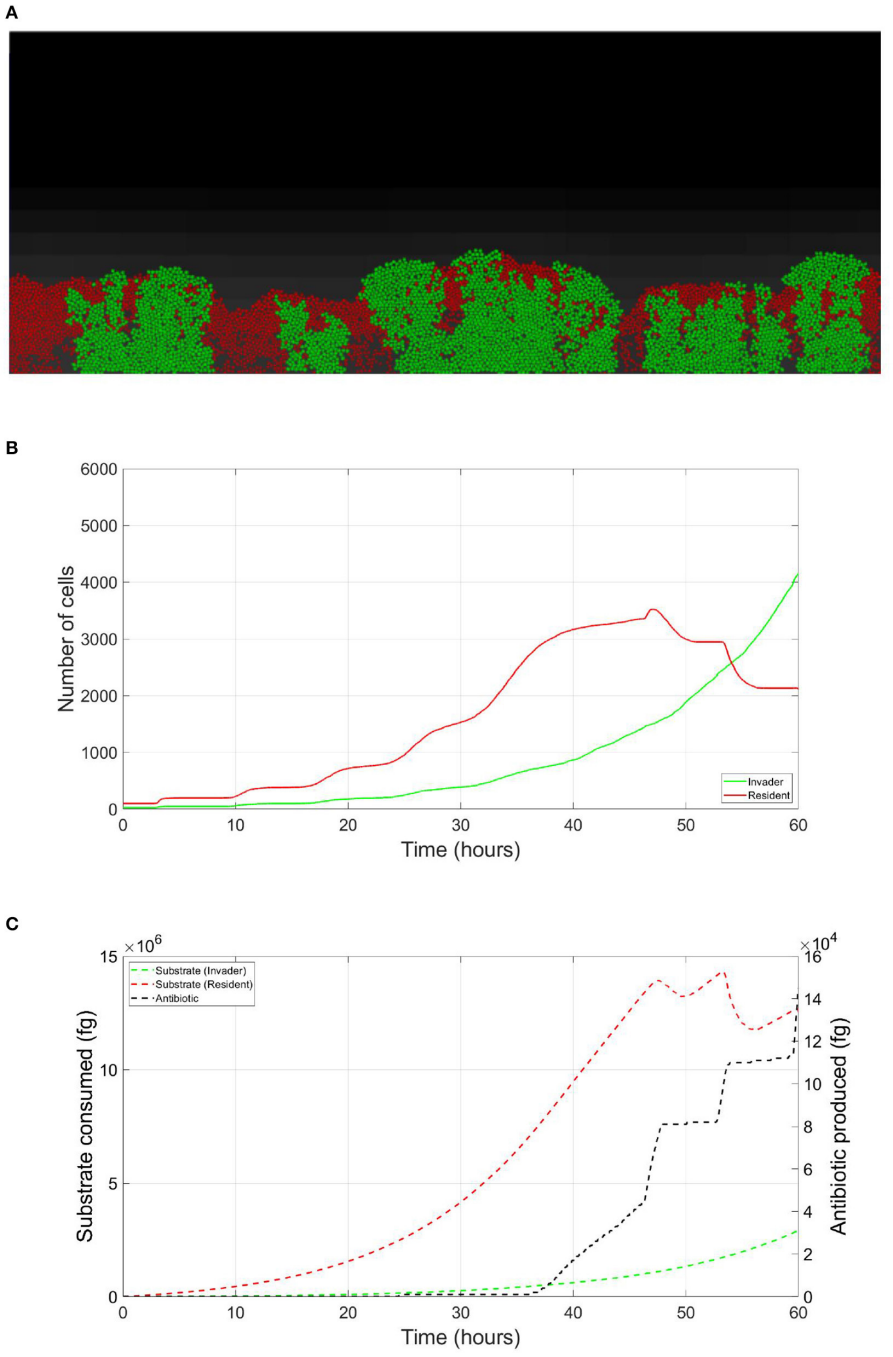
Competition tolerance, no signaling scenario: the usage of a high growth rate/ low yield strategy by the resident species, while being usually an optimal competitive strategy, leads to a quick depletion of nutrients, triggering antibiotic production by the invader species and subsequent decrease in the overall population of both species. **(A)** A snapshot of the competition between the invader species (green) vs. the resident species (red), by the end of the simulation. **(B)** The evolution of the population of an antibiotic producing invader and a resident species, growing on the same nutrient. **(C)** The evolution of the consumption of the nutrient by the invader species (green), and the resident species (red), as well as the production of antibiotic by the invader species (black).

**Figure 3:**
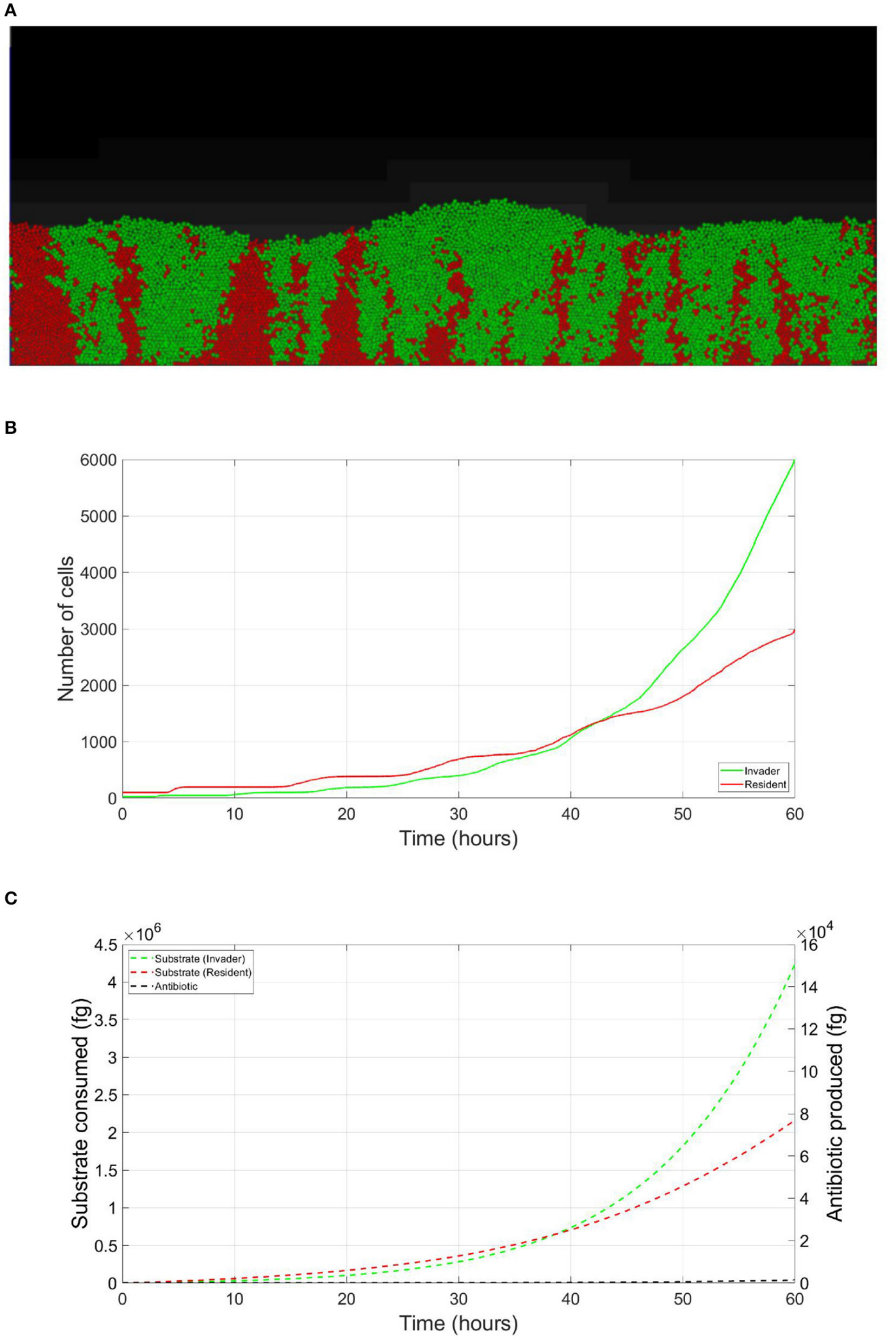
Competition tolerance, signaling scenario: by switching to low growth rate/ high yield strategy, the resident species can achieve more efficient usage of resources, avoiding triggering of the release of the expensive antibiotic by the invader, both parties avoid a costly fight. **(A)** A snapshot of the competition between the invader species (green) vs. the resident species (red), by the end of the simulation. **(B)** The evolution of the population of an antibiotic producing invader and a resident species, growing on the same nutrient, with the resident species adopting low growth rate/ high yield strategy. **(C)** The evolution of the consumption of the nutrient by the invader species (green), and the resident species (red), as well as the production of antibiotic by the invader species (black).

**Figure 4:**
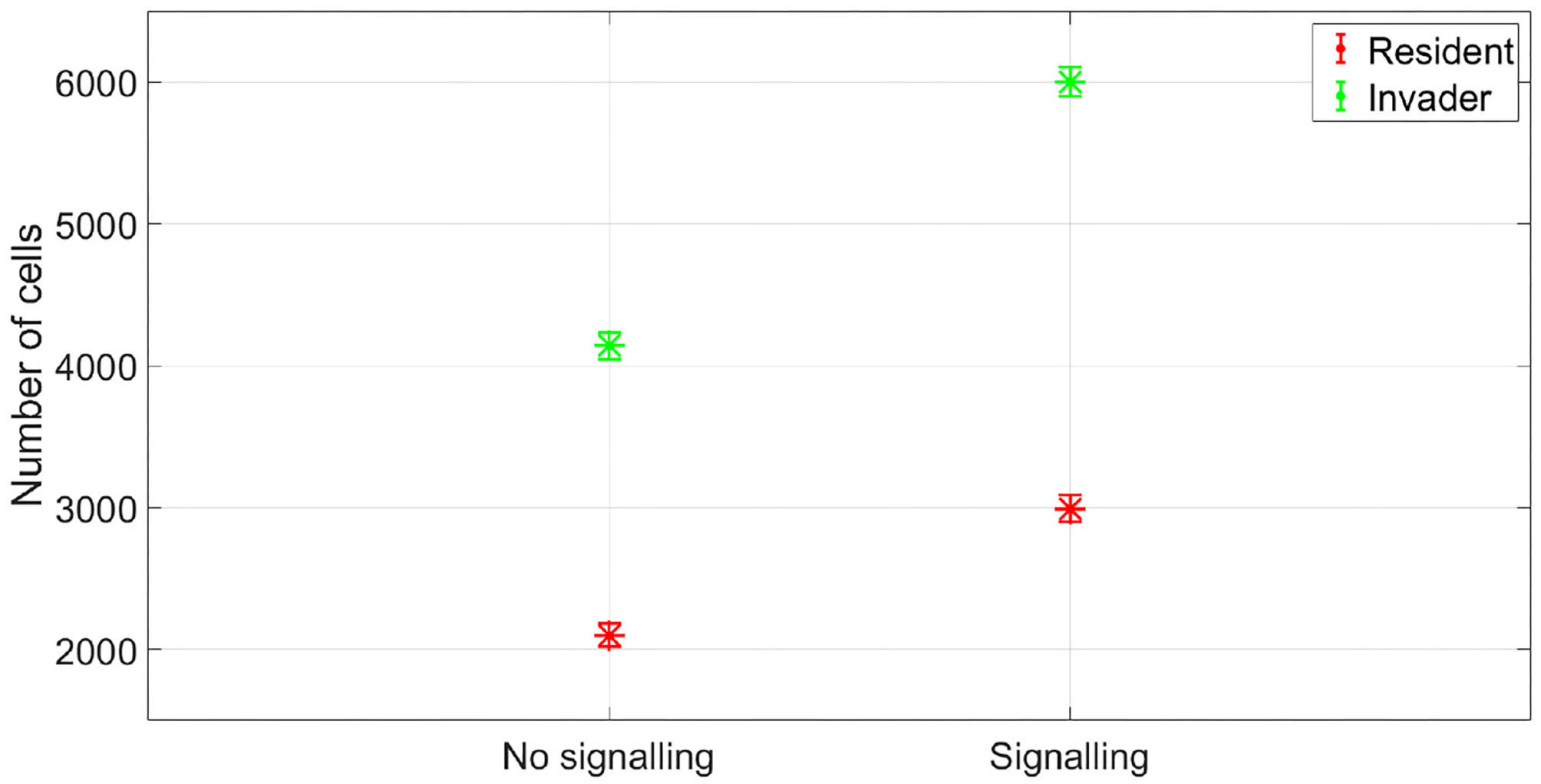
The fitness of the two species in no antibiotic signaling scenario vs. signaling scenario. Again signaling would be expected to be evolutionary stable here as it achieves a gain in fitness for both the sender and receiver. The asterisks represent the mean of the results of 50 simulations, while the error bars represent the standard deviation of the results.

The resident species can still compete in the same niche of an invader, sharing the same resources and spatial space, while not triggering the invader's stress responses. When a resident species is in a competition with an invader whose antibiotic production is regulated by nutrient stress, as depicted in Figure 1, a change in the growth strategy of the resident species could reduce the competitive stress on the invader. The growth strategy of a bacterial species can be defined as the combination of its growth rate and yield (Kreft and Bonhoeffer, 2005; Lipson, 2015). It is well–documented that trade-offs exist between the two values in microbial metabolism (Westerhoff et al., 1983; Lipson, 2015; Wortel et al., 2018), giving rise to a “Pareto front” of optimal growth strategies (Jadot et al., 1996, 1998; Lipson, 2015). These strategies can be broadly classified into two classes. High growth rate/ low yield strategies, where bacteria enjoy fast growth rate at the expense of an inefficient use of resources, and low growth rate / high yield strategies, which are characterized by a slow growth rate with more efficient utilization of resources (Lele and Watve, 2014; Bachmann et al., 2016; Ramin and Allison, 2019). Hence, these two classes are a representation of the r and K ecological strategies, respectively, (Southwood et al., 1974). For example, *Holophaga foetida* can switch from low growth rate/ high yield strategy to high growth rate/ low yield strategy by switching its catabolism to lower ATP yield, resulting in doubling its maximum specific growth rate at the expense of halving its yield (Kreft and Schink, 1993; Kappler et al., 1997). Additionally, there is a diverse range of biological activities by which a bacterial species can change its growth strategy. For example, producing extracellular enzymes and cytotoxins, instead of fully investing in creating new biomass, are activities that sacrifice the maximum growth rate in order to have a better utilization of resources, resulting in a higher yield (Ramin and Allison, 2019). Low growth rate/ high yield strategies lead to a more efficient utilization of the available resources, which is most advantageous when nutrients are scarce. However, when competing over resources with a fast growing competitor, high growth rate/ low yield strategies are more successful (Lipson, 2015). That is except if this competitor has a nutrient stress regulated antibiotic expression, as shown in Figure 2. When the resident species uses a high growth rate/ low yield strategy, the antibiotic production is initiated leading to a drop in the biomass of the resident species. On the other hand, in Figure 3, when the resident species switches to a low growth rate/ high yield strategy, its nutrient consumption is reduced, avoiding the triggering of the stress responses of the invader, resulting in a higher fitness for both the invader and resident species compared to the first case. Here again, both the invader and the resident species benefit when the resident species signal its ability to produce the antibiotic, see Figure 4.

### 3.2. Niche Segregation

**Figure 5:**
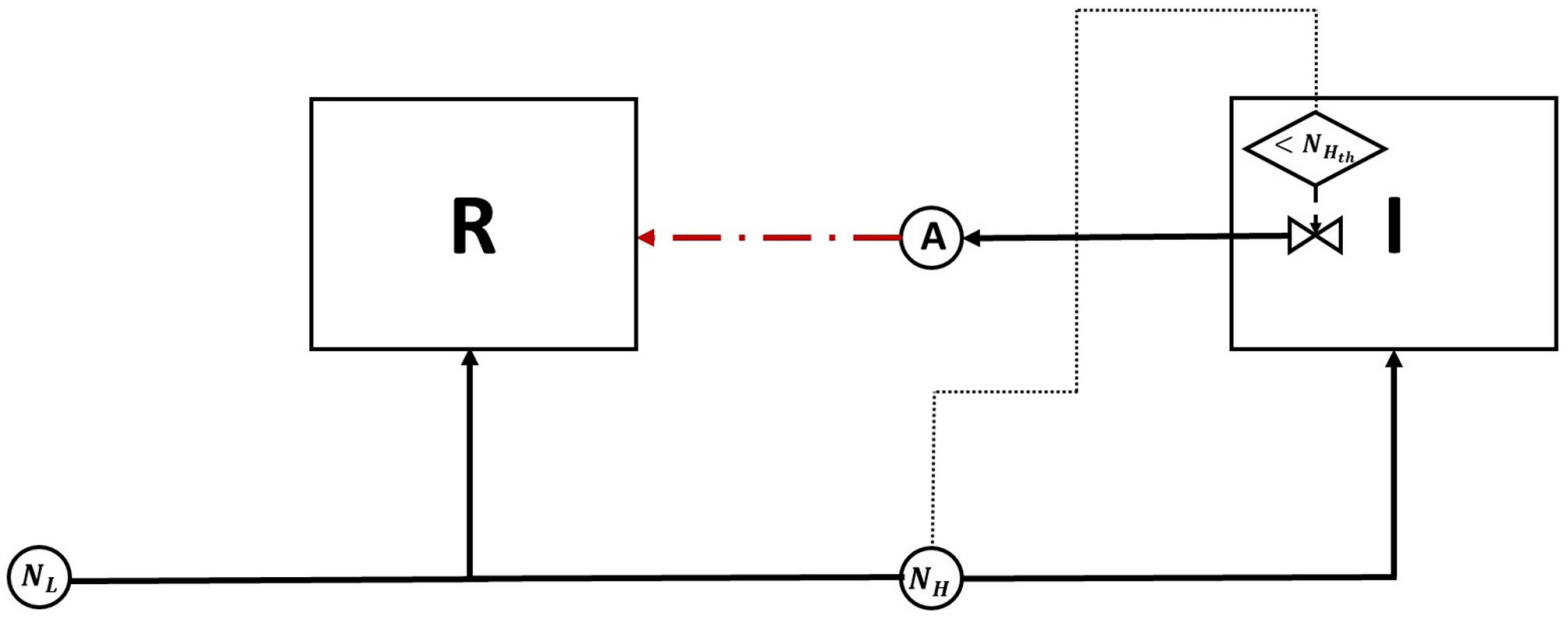
A diagram of the competition between an antibiotic producing invader and a resident species, competing over a high value nutrient, *N*_*H*_. While consuming the high value nutrient is the optimal growth strategy for the resident species in absence of a antibiotic producing opponent, niche segregation by switching to consuming the low value nutrient, *N*_*L*_, is the optimal response when competing with such opponent, as it would avoid triggering nutrient stress regulated antibiotic release by the invader. See Figure 2 legend for an explanation of the diagram's symbols.

**Figure 6:**
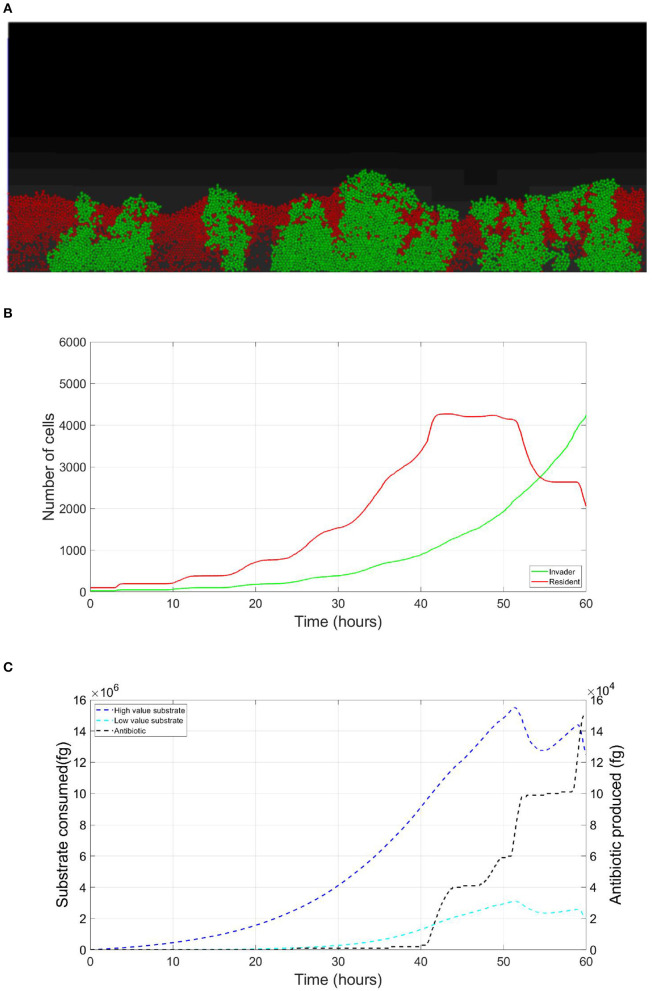
Niche segregation, no signaling scenario: in absence of signaling between the invading and resident species, the competition over the same high value nutrient leads to triggering nutrient stress antibiotic release, which damages the population of the resident species at a cost to the invader species. **(A)** A snapshot of the competition between the invader species (green) vs. the resident species (red), by the end of the simulation. **(B)** The evolution of the population of an antibiotic producing invader and a resident species, competing over a common resource. **(C)** The evolution of the total consumption of the two nutrients as well as the production of the antibiotic by the invader species.

**Figure 7:**
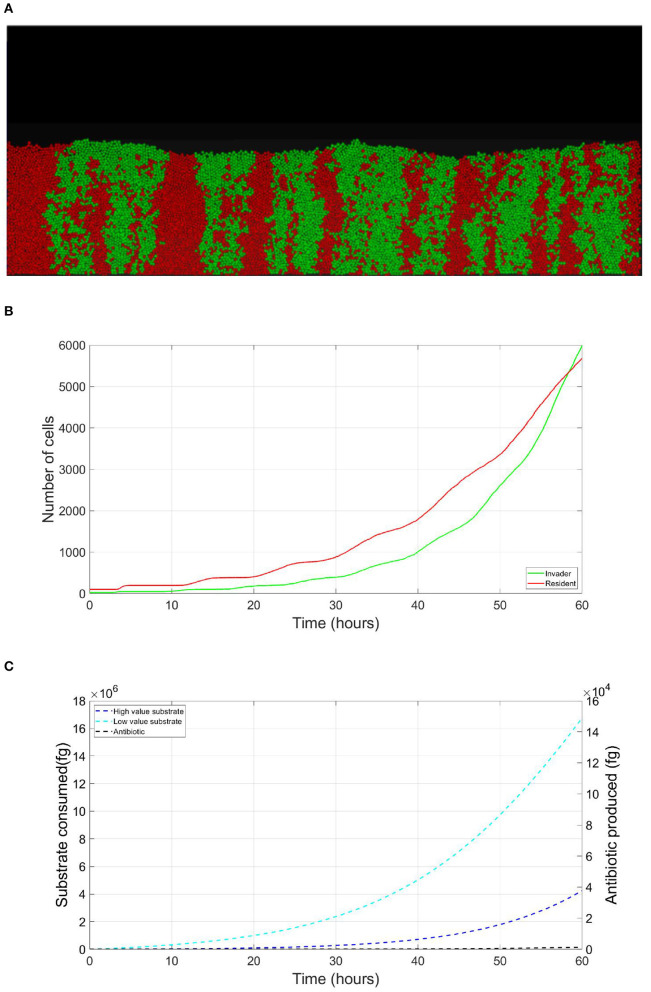
Niche segregation, signaling scenario: when the resident species responds to an antibiotic signal from the invader species by switching to its metabolism to a low value nutrient, the further release of the antibiotic is avoided, to the benefit of both species. **(A)** A snapshot of the competition between the invader species (green) vs. the resident species (red), by the end of the simulation. **(B)** The evolution of the population of an antibiotic producing invader and a resident species, when the resident species respond to signaling by specializing in the low value nutrient. **(C)** The evolution of the total consumption of the two nutrients as well as the production of the antibiotic by the invader species.

**Figure 8:**
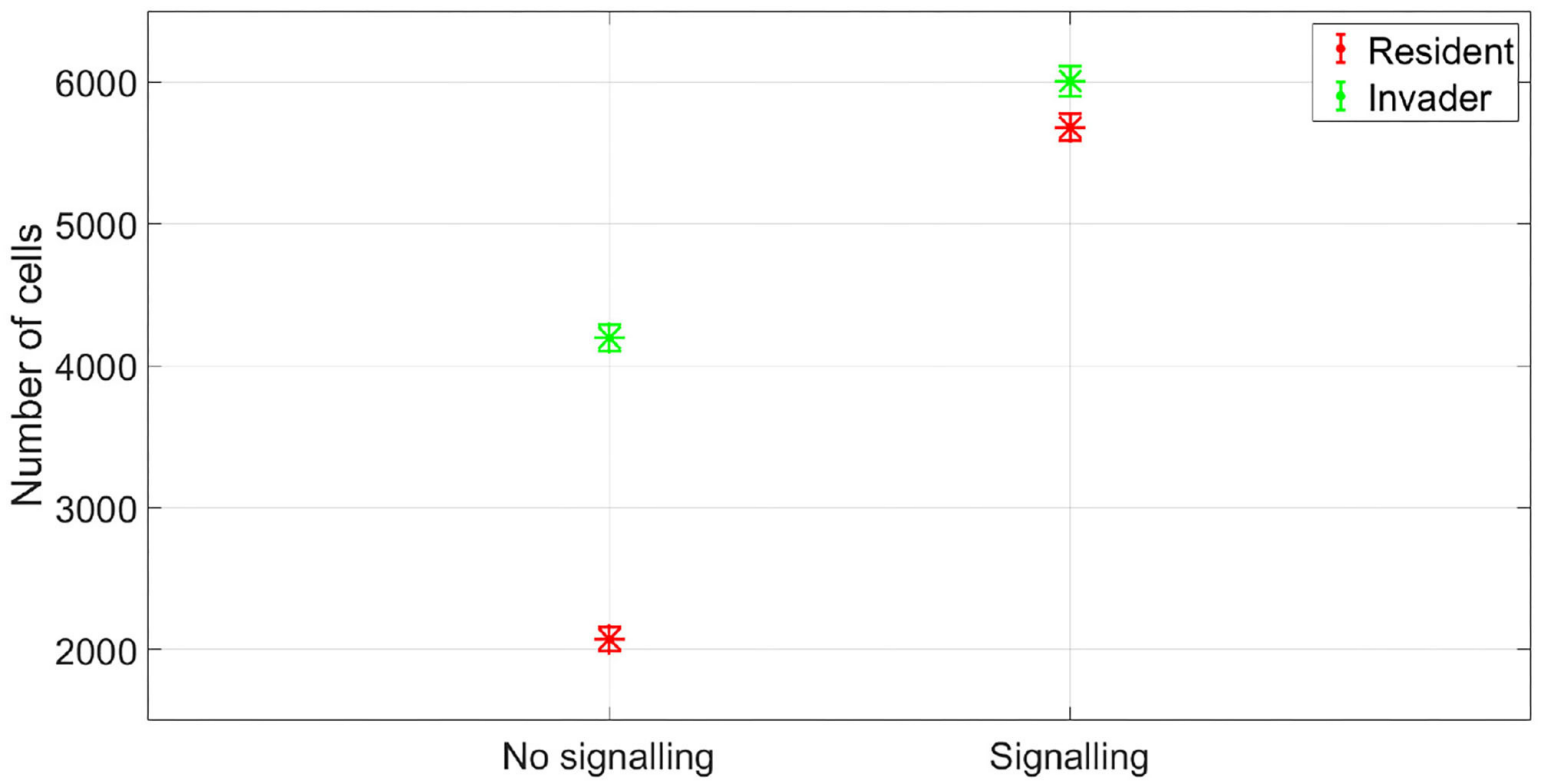
The fitness of the two species in no antibiotic signaling scenario vs. signaling scenario. In the signaling scenario, both the invader and the resident species benefit from the evolution of a signaling mechanism. The data representation is the same as Figure 4.

Niche segregation refers to the resident species reducing the niche overlap by switching to underutilized nutrients when the depletion of the contested, high value, resource could trigger antibiotic production by the invading species. Those underutilized nutrients could be ones that give rise to lower growth rates compared to the contested resource, and hence, are less likely to justify an investment in antibiotic production compared to the original high value resource. A model of such situation is presented in Figure 5. Here, the resident species and the invader species can consume two nutrients, a high value nutrient *N*_*H*_, on which the bacteria can achieve a maximum growth rate of μ_*max,high*_, and a low value nutrient *N*_*L*_ which can sustain a maximum growth rate of μ_*max,low*_, with μ_*max,low*_ < μ_*max,high*_. The growth of a microorganism on multiple substrates has been studied by Monod (1949). The best growth strategy of a microbial species in absence of competition or in competition with a non-antibiotic producing invader is to metabolize the high value nutrient first, the one which gives rise to the fastest growth rate (Monod, 1949; Kompala, 2013). However, when competing with an invader which initiates antibiotic production under nutrient stress, as shown in Figure 6, the resident species incurs significant damage. As the nutrient concentration gets too low, antibiotic production by *I* is initiated, leading to a significant reduction in *R* biomass and freeing more resources for *I*. However, if *R* responded to signaling by *I* with changing its growth strategy to solely consume *N*_*L*_, the nutrient stress response of *I* will not be activated, resulting in a higher fitness for both *I* and *R* compared to the “standard” growth strategy of *R*, see Figure 7. This means that both *R* and *I* benefit from an alteration of the growth strategy of *R* when *I* is capable of antibiotic production, see Figure 8, which makes signaling beneficial for the two species.

### 3.3. Signals' Honesty

**Figure 9:**
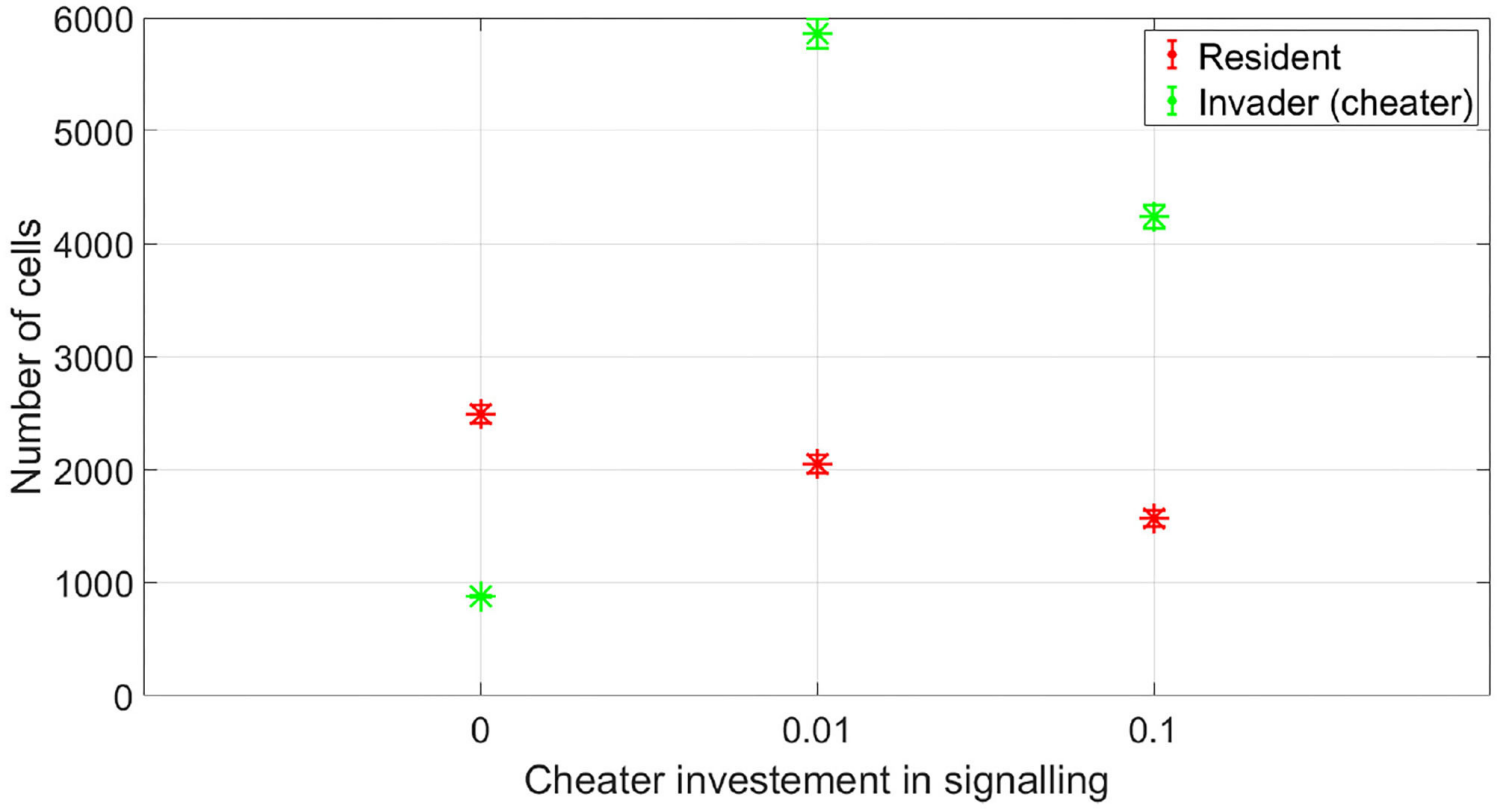
The fitness of a non-efficient antibiotic producer vs. the resident species, when the resident species adopts a competition tolerance strategy at a low subinhibitory antibiotic concentration. While the non-efficient antibiotic producer is not capable of inflicting significant damage at the resident species, it can produce enough quantities of the antibiotic to activate the competition tolerance response of the resident species, achieving a high fitness gain in the process. The data representation is the same as Figure 4.

**Figure 10:**
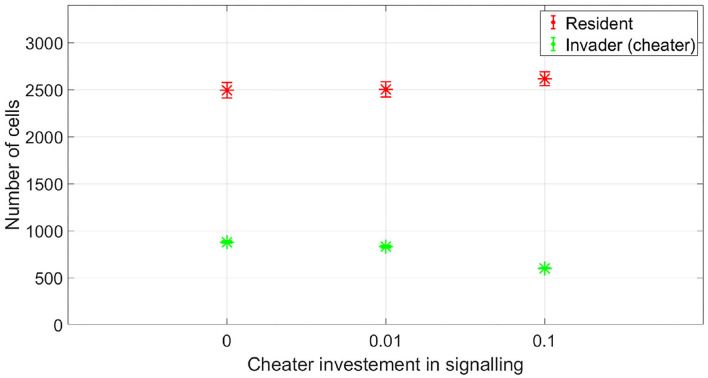
The fitness of a non-efficient antibiotic producer vs. a resident species, when the resident species adopts a competition tolerance strategy at a high subinhibitory antibiotic concentration. By setting a high signal threshold, the resident species avoid abusing its competition tolerance response by weak opponents. Here, the cost incurred by the non-efficient antibiotic producer to activate the competition tolerance response is higher than the potential gain. The data representation is the same as Figure 4.

**Figure 11:**
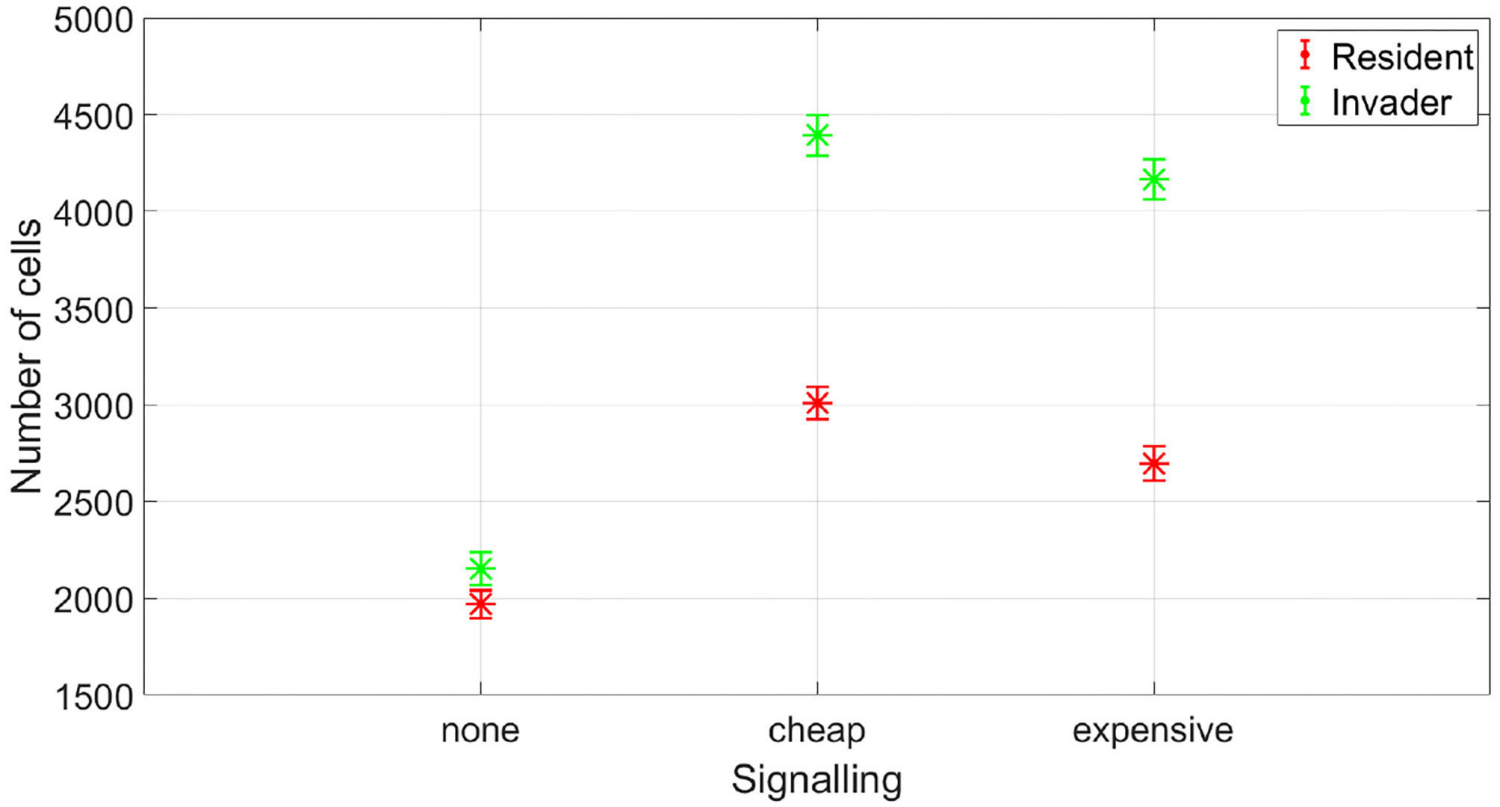
The fitness of an efficient antibiotic producer vs. a resident species, at different signaling scenarios. While expensive signaling is costly for both the resident and the invader species, it ensures honest communication. The data representation is the same as Figure 4.

At which SIC should the resident species activate its competition tolerance/ niche segregation response? The disadvantage of activating the response at very low concentrations is that it could be taken advantage of by cheaters, strains that are not capable of inflicting real harm on the resident species, but could still produce enough antibiotic to activate a response of the resident species that reduce its competitiveness. To illustrate this, we simulated the competition between a resident species which stops producing its own antibiotic at low SIC and an invading species which is not efficient at producing antibiotic. This is simulated by setting its stoichiometric coefficient for antibiotic production to α = 0.4 (mg antibiotic/mg bacteria), instead of the nominal value, α = 4 (mg antibiotic/mg bacteria). In Figure 9, it is shown that while the resident species is able to outperform the invader species in absence of signaling, the invader species can take advantage of the competition tolerance response of the resident species and outgrow it. Here, the invader species do not have the capacity to beat the resident species by producing antibiotics. However, it has the capacity to produce antibiotics at a concentration that could be detected by the resident species which responds to that by reducing its competitive impact. The resident species could avoid being exploited by inefficient antibiotic producers by raising the concentration at which it responds to the signal. As shown in Figure 10, when utilizing a high concentration to respond to the signal, 0.04 mg/l instead of 0.01 mg/l, the cheater species do not achieve a fitness gain by investing in antibiotic production to activate the competition tolerance mechanism of the resident species. That is because at high concentration, the fitness advantage gained by the invader species by exploiting the resident species is outweighed by the cost of producing such expensive signal. As we see here, the communication system's reliability increases with the cost of signaling, in accordance with the predictions of the signaling theory. Finally, Figure 11 summarizes the outcomes of competition between an efficient antibiotic producer and a resident species in absence of signaling, a cheap signaling system, and an expensive signaling system, respectively. It is seen here that while the usage of both signaling systems is beneficial to both parties compared to the no signaling scenario, the expensive signaling system incurs an additional cost on both the receiver and the producer of the signal, compared to the cheap one. However, since the signal receiver is the one setting the cost of the system, the expensive signaling system will be beneficial to the signal receiver as it can avoid being exploited by cheaters. In brief, if the activation concentration is too low, even an inefficient antibiotic producer can activate it, leading to an increase in the fitness of the invader and a drop in the fitness of the resident compared to the non-signaling case. However, if the activation concentration is high enough, it will be expensive to a non-efficient antibiotic producer to activate the competition tolerance mechanism of the resident species. Hence, the activation concentration set by the resident can act as a way to filter opponents by strength, ensuring that the competition tolerance/ niche segregation growth mechanisms are only activated in the presence of real danger.

### 3.4. The Evolution of a Weapon Into a Signal

A final question that we would like to briefly discuss is *how* can a weapon turn into a signal. Explaining the evolution of a communication system in nature is not straightforward, and it has been described as a chicken and an egg problem as signals and responses are mutually dependent on each other (Scott-Phillips et al., 2012). We hypothesize that antibiotics acquired their signaling function via the process of ritualization (Maynard-Smith et al., 2003; Bradbury and Vehrencamp, 2011; Rowe et al., 2018). Here, the signaling system evolves initially from cues that the receiver could take advantage of. In our case, we propose that the evolution of antibiotics as signaling molecules happened through two steps. First, a selection on the resident species which could detect and respond to cues from antibiotics-releasing invader species. The resident species which are most successful at detecting those cues, and consequently the antibiotic producing species at their neighborhood and manage to avoid triggering their stress responses, will get a fitness advantage over their peers. The following step is selection of the invader species based on how easy they can be detected by potential resident species. The invader species that could leave more easily detected “cues,” produce a detectable concentration of antibiotics, could be distinguished by a higher fraction of resident species, therefore avoiding costly conflicts and gaining a fitness advantage in the process. Through these two steps, which have a positive feedback effect on each other, both components of the communication system, resident species that can detect antibiotic concentration and act to reduce the competitive impact and invader species that produce detectable amounts of antibiotics, can both evolve. It is noted as well that due to diffusion constraints acting within a biofilm; an antibiotic, when used as a weapon, will exist at a high concentration in the direct neighborhood of the producer cells but dilutes forming a gradient away from them (Baquero et al., 2021). Therefore, in the periphery of this gradient the antibiotic concentration would not be sufficient to produce an inhibiting effect but can still serve as a cue to the recipient cells. Consequently, this could provide an alternative path for the evolution of a signaling system between the antibiotic producer and the recipient cells.

## 4. Conclusion

For a signal to evolve between two distinct species, there have to be benefits for both the receiver of the signal and the sender of the signal. Otherwise, the signal will not be evolutionary stable. In this paper, we hypothesize that owing to its original function as a weapon, an antibiotic can serve as a signal as well. Antibiotics are both expensive to produce and quite damaging to ecological competitors. Hence, it is both in the interests of the producer and recipient of antibiotics to avoid its usage. By evolving mechanisms to reduce the competitive impact, such as switching to less valuable nutrient, as it has been experimentally documented in Jauri et al. (2013), or moving to a less competitive growth strategy, the triggering of the stress responses required for the release of the antibiotics can be avoided, hence reaching a more favorable outcome for both species. The view of the antibiotics as weapons has been motivated by a picture of a violent, extremely competitive, microbe-kill-microbe world. On the other hand, the proponents of the antibiotics as signals view the microbial society as a more cooperative one. Our work paints an in-between picture of the microbial world in which conflicts arise, weapons can and will be used, but also conflicts can be reduced via signaling. This view of the nature of the microbial conflict is aligned with how weapons are used in human society and between animals. However, while most animals have relatively sophisticated brains, our work shows that even much more simpler decision systems that are commonly found in bacteria can still be capitalized on to provide a peaceful resolution of frequent conflicts. The language of force is said to be universal and it could be one that bacteria commonly use. Our work suggests a way to exploit this communication mechanism between bacterial species. By targeting the signal response systems at the recipient bacteria to antibiotics, one would expect that this will lead to higher chance of “civil wars” among a microbial society, which could be beneficial from a human perspective.

## Data Availability Statement

The raw data supporting the conclusions of this article will be made available by the authors, without undue reservation.

## Author Contributions

IH and JV: conceptualization, resources, writing, and review. IH: investigation, literature review, writing, and original draft preparation. All authors contributed to the article and approved the submitted version.

## Funding

This work was supported by the KU Leuven Research Council (OPTEC Center-of-Excellence Optimization in Engineering OPTEC and project C24/18/046), by the ERA-NET FACCESurPlus FLEXIBI Project, co-funded by VLAIO project HBC.2017.0176, by the Fund for Scientific Research-Flanders (projects G.0863.18 and G.0B41.21N), and by the European Union's Horizon 2020 Research and Innovation Programme (Marie Sklodowska-Curie grant agreement numbers 813329 and 956126).

## Conflict of Interest

The authors declare that the research was conducted in the absence of any commercial or financial relationships that could be construed as a potential conflict of interest.

## Publisher's Note

All claims expressed in this article are solely those of the authors and do not necessarily represent those of their affiliated organizations, or those of the publisher, the editors and the reviewers. Any product that may be evaluated in this article, or claim that may be made by its manufacturer, is not guaranteed or endorsed by the publisher.
